# Interfacial Properties of Active-Passive Polymer Mixtures

**DOI:** 10.3390/e20070520

**Published:** 2018-07-10

**Authors:** Jan Smrek, Kurt Kremer

**Affiliations:** Max Planck Institute for Polymer Research, Ackermannweg 10, 55127 Mainz, Germany

**Keywords:** active matter, polymers, capillary waves

## Abstract

Active matter consists of particles that dissipate energy, from their own sources, in the form of mechanical work on their surroundings. Recent interest in active-passive polymer mixtures has been driven by their relevance in phase separation of (e.g., transcriptionally) active and inactive (transcriptionally silent) DNA strands in nuclei of living cells. In this paper, we study the interfacial properties of the phase separated steady states of the active-passive polymer mixtures and compare them with equilibrium phase separation. We model the active constituents by assigning them stronger-than-thermal fluctuations. We demonstrate that the entropy production is an accurate indicator of the phase transition. We then construct phase diagrams and analyze kinetic properties of the particles as a function of the distance from the interface. Studying the interface fluctuations, we find that they follow the capillary waves spectrum. This allows us to establish a mechanistic definition of the interfacial stiffness and its dependence on the relative level of activity with respect to the passive constituents. We show how the interfacial width depends on the activity ratio and comment on the finite size effects. Our results highlight similarities and differences of the non-equilibrium steady states with an equilibrium phase separated polymer mixture with a lower critical solution temperature. We present several directions in which the non-equilibrium system can be studied further and point out interesting observations that indicate general principles behind the non-equilibrium phase separation.

## 1. Introduction

Active matter provides a playground to study non-equilibrium statistical physics and self-organizing properties of living systems [1,2]. The constitutive ingredients are the active particles which, unlike in thermal equilibrium, constantly consume and dissipate energy to propel or perform mechanical work. Because a unifying description of these non-equilibrium systems is missing, it is important to explore the properties of simple models and their relation to equilibrium statistical physics.

Two classes of simple activity models can be determined. The prominent *vectorial* class, where the particles are propelled in a given direction that randomizes through various mechanisms on longer time scales, includes Viscsek model, Active Brownian particles, Run and Thumble, and Ornstein–Uhlenbeck particles [3,4,5,6,7]. The simpler and less explored *scalar* activity, on which we focus in this work, is modeled as thermal-like fluctuations of elevated temperature with respect to the passive surroundings. Therefore, it is defined in mixtures of active and passive particles [8,9].

It was shown that a sufficiently high temperature ratio of the two particle species results in a phase separated steady state [8,9]. While the critical ratio is unfeasibly high for colloidal particles, if the particles are polymers, the ordered phase may be experimentally realized, as the critical ratio decreases with their length [10]. This supports the hypothesis that the effect might play a role in separation of active and passive DNA strands in living cells [9,10,11,12]. There, the activity can originate from transcription, repair or looping extrusion processes that consume energy to fuel the molecular motors that drag the DNA strands. For example, it was observed that the euchromatin, the transcriptionally active DNA fiber, is spatially separated from the mostly transcriptionally silent heterochromatin. As shown in [13], some data on the dynamics of the active DNA fibers in living cells can be understood by such a simple two-temperature model.

The heat transport and related entropy production both take place at the interface and govern the system’s behavior [10]. Investigating the interface of the phase-separated steady state can help to reveal not only the underlying principles of the non-equilibrium phase transition, but also new phenomena. For example, there are indications that a spontaneously separated *vectorial* active matter exhibits similar interfacial fluctuation spectra as the equilibrium phase separated state [14,15,16]. However, unlike in equilibrium, positive interfacial stiffness arises as a competition between negative interfacial tension and the work of oriented active particles stabilizing the interface. These features are hypothesized to be a generic characteristic of activity induced phase separated matter, but it is not clear if this applies to scalar activity blends as well. Comparing the interfacial properties of the non-equilibrium steady states with equilibrium phase-separated systems enables delineation of where the physical understanding built on the equilibrium systems is applicable.

In this work, we use molecular dynamics (MD) to study the interfacial properties of a well-separated steady state of a blend of two identical polymer types connected to two different thermostats. First, we describe the model details. Subsequently, we characterize the interface profiles, derive the phase diagrams, explore the critical behavior, and show that the entropy production is an accurate indicator of the phase transition. A significant part of our study is dedicated to interface fluctuations. We characterize their spectrum, which allows for a mechanistic definition of the interfacial stiffness. The various results point to a common conclusion that this non-equilibrium system resembles in many respects an equilibrium polymer blend with a lower critical solution temperature.

### Model

Our system consists of *M* monodisperse fully flexible chains of N=40 monomers with purely repulsive shifted Lennard–Jones interactions with a cutoff at $r_{c}=2^{1/6}\sigma$

(1) $$U_{\mathrm{LJ}}\left(r\right)=4\varepsilon \left[{\left(\frac{\sigma }{r}\right)}^{12}-{\left(\frac{\sigma }{r}\right)}^{6}\right]+\varepsilon .$$

The chain connectivity is maintained by the finitely extensible nonlinear elastic (FENE) potential

(2) $$U_{\mathrm{FENE}}\left(r\right)=-\frac{1}{2}r_{\max}^{2}K\log\left[1-{\left(\frac{r}{r_{\max}}\right)}^{2}\right],$$

where $K=30.0\varepsilon /\sigma^{2}$ and $r_{m}=1.5\sigma$ The natural time scale of this standard polymer model is $\tau =\;\sigma {\left(m/\varepsilon \right)}^{1/2}$. All energy quantities are in units of ε and the Boltzmann constant is set to unity.

**Figure 1:**
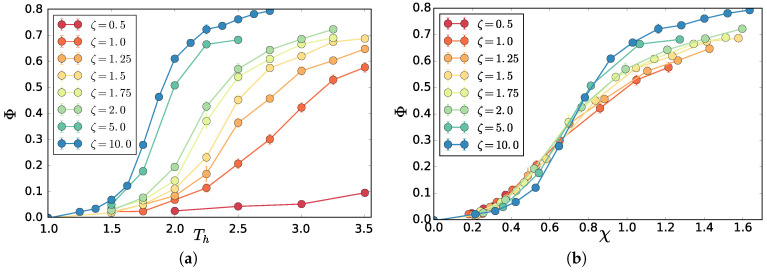
(**a**) order parameter Φ as a function of $T_{h}$ for various coupling frictions ζ in units of $\tau^{-1}$, see the text for the definition of Φ. For well separated regime Φ→1, Φ is below unity for high $T_{h}$ (or χ) due to impure phases and finite size effects. Error bars (usually smaller than the symbol size) are standard deviations over 30 well separated conformations; (**b**) same data as a function of χ shows much smaller variation than for $T_{h}$ .

M/2 of the chains are coupled to a “cold” Langevin thermostat with temperature $T_{c}=1.0$ and the other half to a “hot” Langevin thermostat of $T_{h}>T_{c}$. Each thermostat is coupled to the system by the same coupling friction ζ. In the original work on polymers, Ref. [10] ζ was chosen to be rather high $10\tau^{-1}$ to be comparable to the overdamped limit used in other works [11] and to observe phase separation at relatively low thermostat temperatures. The critical temperature of the hot thermostat decreases with increasing friction (see Figure 1a) because the heat transfer from the thermostat to the particles becomes more efficient, which increases the difference between the effective temperature of the hot and the cold particles. As mentioned in [10], a natural asymmetry parameter of the system is $\chi =\left(T_{h}^{\mathrm{eff}}-T_{c}^{\mathrm{eff}}\right)/T_{c}^{\mathrm{eff}}$ which captures the relative difference in the effective temperatures $T_{h}^{\mathrm{eff}}$ and $T_{c}^{\mathrm{eff}}$ (one third of the mean squared velocity) of the hot and cold components, respectively. We use χ because of its low sensitivity to the choice of friction as shown in Figure 1b, where we compare order parameter profiles for different frictions for the system of M=1000 chains. The order parameter used is Φ=2x−1, where *x* is the average inter-chain like-particles number fraction in a $r_{c}$ neighborhood i.e., if inter-chain cold-cold monomer contacts are equally likely to hot-cold Φ=0. As can be seen in Figure 1b, some systematic variation with ζ still remains, which we leave for future study.

To obtain a well separated state for profile and capillary wave analysis, we start with an equilibrium configuration of a well mixed system at a constant volume setting with density of $0.85\sigma^{-3}$, temperature T=1.0 with periodic boundary conditions. We connect the hot chains to the hot thermostat of $T_{h}\geq 2.25\varepsilon$, which is sufficient for phase separation to occur given the coupling friction $\zeta =1.5\tau^{-1}$ used throughout this work. We simulate the system with time step 0.005τ until the phase separated steady state is reached. This process typically takes about the time needed for all the chains to diffuse through the whole system. Above the critical temperature ratio, the interface aligns with one of the box coordinates as is typical also for the equilibrium phase separation—there, due to the minimization of the interfacial area. For our largest system of M=5000, the aligned steady states were observed for at least $2\times 10^{6}\tau$. We sampled the configurations every 5000τ, which is enough time to get uncorrelated chain conformations. We had at least 400 configurations for each $T_{h}$ in total.

To characterize the steady state, we will use various averages of the physical quantities. The brackets 〈…〉 stand for average over the ensemble of the configurations in the steady state and this represents an average over subset of the phase space that characterizes the steady state. For certain quantities, such as the density, the average 〈…〉 can vary with distance from the interface. In that case, the average at a given distance is calculated over the steady state configurations of the particles that fall within a given slab at that distance.

## 2. Profiles and Phase Diagrams of Density and Effective Temperature

**Figure 2:**
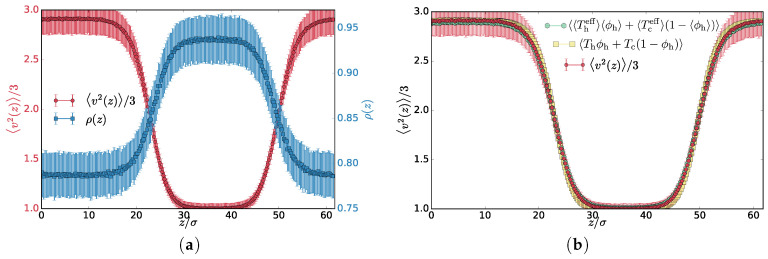
(**a**) average density and msv profiles for $T_{h}=3.0$; (**b**) comparison of the profiles of the msv (red circles), msv calculated from the effective temperatures $T_{h}^{\mathrm{eff}}\phi_{h}+T_{c}^{\mathrm{eff}}\left(1-\phi_{h}\right)$ (green hexagons) and the approximation to msv computed as $T_{h}\phi_{h}+T_{c}\left(1-\phi_{h}\right)$ (yellow squares) for $T_{h}=3.0$.

The phase separation occurs because the local difference in temperatures gives rise to a pressure difference that is compensated with a density change by the redistribution of the particles. In steady state, the mass transport is suppressed, and heat flow is mostly localized at the interfaces. Therefore, in a separated steady state, the effective temperature and density exhibit non-constant profiles, while the pressure tensor component perpendicular to the interface is constant (hydrostatic equilibrium ∇·p=0). To investigate the profiles, we orient the interface perpendicular to the *z*-axis and align the different system snapshots by subtracting the center of mass from the coordinates. The average density exhibits a two-phase profile with two interfaces due to the periodic boundary conditions, as can be seen in Figure 2a for $T_{h}=3.0$. We illustrate other properties of the system in figures with $T_{h}=3.0$, but we measured the same properties for all the examined temperatures $T_{h}$ .

To examine the kinetic properties of the particles, we compute the following quantities: (i) $\langle v^{2}\left(z\right)\rangle /3$ is the mean square particle velocity (msv) over all particles in a slab at position *z* and (ii) $T_{h}^{\mathrm{eff}}\left(z\right)$ and $T_{c}^{\mathrm{eff}}\left(z\right)$, which is one third of the mean square velocity of only the hot and cold particles in the slab, respectively. We found that for each *z* the $T_{h}^{\mathrm{eff}}\left(z\right)$ and $T_{c}^{\mathrm{eff}}\left(z\right)$ have (two different) approximately Maxwell distributions and therefore represent effective temperatures for the subsets of particles in a given slab. The only deviation from the Maxwell distribution occurs very close to the interface, which we discuss later below. The msv ${\langle v^{2}\rangle }_{z}/3$, on the other hand, is a mean over all particles in a slab and therefore is Maxwell distributed only if the slab contains just a pure phase.

**Figure 3:**
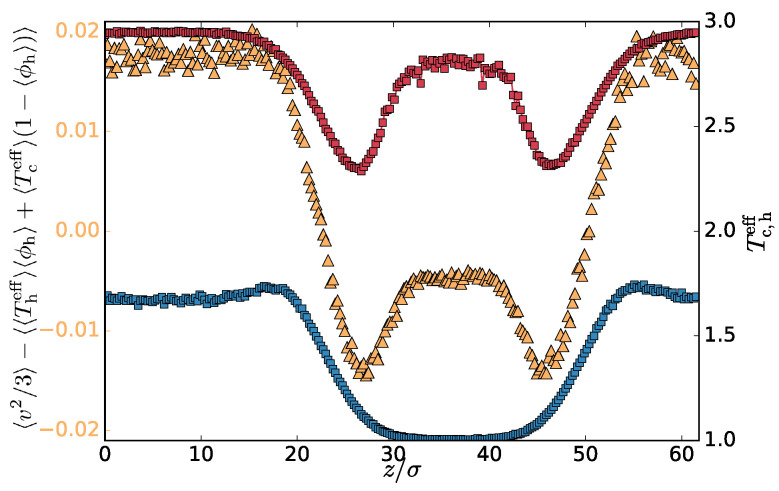
Left axis (yellow triangles): Profile of the difference between the msv and the effective temperature for $T_{h}=3.0$. Right axis (circles): profiles of the effective temperatures $T_{h}^{\mathrm{eff}}\left(z\right)$ (red) and $T_{h}^{\mathrm{eff}}\left(z\right)$ (cold).

**Figure A2:**
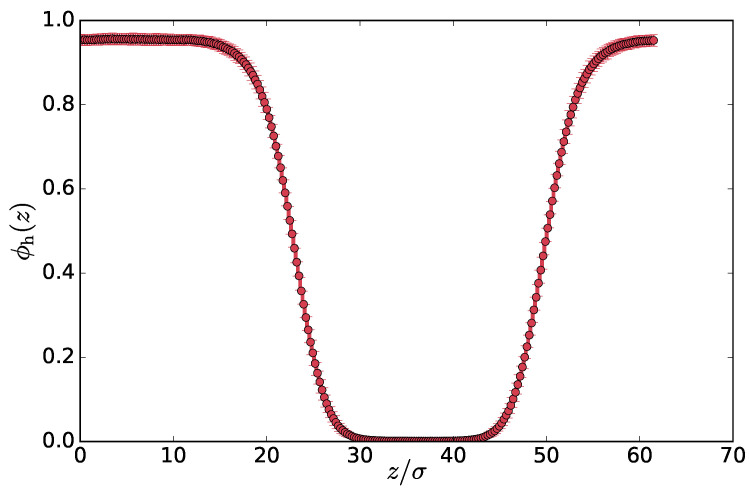
Average profile of hot particles number fraction for $T_{h}=3.0$ computed by Equation (3) and averaged over the steady state configurations.

The less dense hot phase exhibits lower msv than $T_{h}$ because it contains some cold chains as well (see Figure A2). The msv profile is in fact effective temperatures weighted by the number fractions of the components: $\langle v^{2}\rangle /3=\langle T_{h}^{\mathrm{eff}}\phi_{h}+T_{c}^{\mathrm{eff}}\left(1-\phi_{h}\right)\rangle$ computed in each slab and averaged over the different system steady state configuration, with $\phi_{h}=n_{h}/\left(n_{h}+n_{c}\right)$ being the number fraction of the hot monomers. One can also compute the average profiles of the number fractions and the effective temperature and calculate the profile of the combined quantity $\langle \langle T_{h}^{\mathrm{eff}}\rangle \langle \phi_{h}\rangle +\langle T_{c}^{\mathrm{eff}}\rangle \left(1-\langle \phi_{h}\rangle \right)\rangle$. Both of these quantities are plotted in Figure 2b. Their difference (Figure 3) captures the correlation of the number fraction of the hot monomers and the msv. These quantities are positively correlated in the hot phase, mostly uncorrelated in the transition region, and negatively in the cold phase. This is expected since increasing the effective temperature of the hot subset should favour further segregation, increasing the hot number fraction. The correlation profile follows the effective temperature of the hot subset of the particles. Interestingly hot particles are hotter deep inside the cold phase than in the adjacent transition region and, similarly, cold chains are slightly colder in the hot phase than in the transition region (Figure 3). To avoid excessive heat transfer, the conformation of the hot chain in the cold phase is more compact, increasing self-contacts. We confirm this by calculating the mean square gyration radius of the chains in the different regions (Table 1). Analogous effect is observed also in equilibrium phase-separated blends, where the chains of the minority phase are more compact to decrease the unfavourable contacts with the chains of the majority phase.

In fact, deep in the bulk phase, the number fraction of hot monomers $\phi_{h}$ and the msv are related by $T_{\mathrm{eff}}=T_{h}\phi_{h}+T_{c}\left(1-\phi_{h}\right)$ (see Figure 2). This is a consequence of a local flux conservation because there is no heat transport out of the region deep in the bulk phase. In the interfacial region, this relation is slightly violated, as there is a net heat transport to the surrounding areas.

To understand the directional and ensemble properties of the profiles deeper, we investigate the pressure tensor

(3) $$p_{ij}\left(z\right)=\rho \left(z\right)\langle v_{i}\cdot v_{j}\rangle /3+\langle \sum_{n\neq m}{\left(r_{nm}⊗F_{nm}\right)}_{ij}\rangle /2A.$$

This is derived from mechanics considerations only [9,17] and in the non-equilibrium case should be interpreted in the mechanical sense. For example, the trace of the tensor represents the mechanical force per unit area that the box experiences by the presence of the polymer mixture. Our particles do not have any directionality and therefore we do not need to consider any additional “active” terms that appear for vectorial activity class models [14,18]. The averaging in Equation (3), as before, represents an average over the configurations characterizing the steady state.

**Figure 4:**
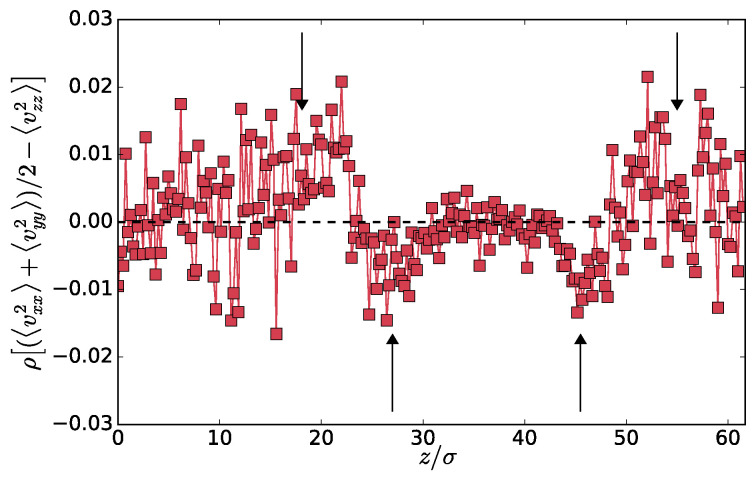
Difference of the tangential and normal components of the kinetic part of the pressure tensor for $T_{h}=3.0$. The dashed line is a guide to the eye for zero value. Arrows correspond to the locations of maxima (minima) of the $T_{c}^{\mathrm{eff}}\left(z\right)$ ($T_{h}^{\mathrm{eff}}\left(z\right)$) shown in Figure 3.

First, in Figure 4, we plot the profile of the difference between mean tangential and normal components of the kinetic part of the pressure tensor: $\rho \left(z\right)\langle \left(v_{xx}^{2}+v_{yy}^{2}\right)/2-v_{zz}^{2}\rangle \left(z\right)$. Although the data is a bit noisy, as we are now using both particles types, we can still observe a non-monotonous profile similar to the one shown in Figure 3. This is particularly interesting in the context of the interfacial tension. In equilibrium phase separation, the interfacial tension γ can be computed as

(4) $$\gamma =\frac{1}{2}\int_{0}^{L}\left[p_{N}-p_{T}\right]dz,$$

where the $p_{N}$ and $p_{T}$ are the normal and tangential components of the pressure tensor. The kinetic part of the pressure tensor does not contribute to γ because it is constant and cancels out from the two components. Interestingly, if an analogous definition of γ applies to the present non-equilibrium system, the non-monotonous profile in Figure 4 shows that the kinetic part of the pressure tensor might contribute to γ. An analogous definition of the surface tension seems to be applicable in the vectorial activity phase separation [14,15].

Figure 4 also shows that, very close to the interface, the distribution of the velocities is not isotropic. This also means that the velocity distributions of the hot or cold particles separately can not be Maxwellian at the interface because the *z*-component has different mean squares than the other two. In the mixed phase, it was found [10] that the two particle types preserve Maxwellian distributions. It is therefore the interface that breaks the rotational symmetry even in the momentum space, which is a pure non-equilibrium effect. The off-diagonal components of the kinetic part of the tensor are within our accuracy zero on average.

**Figure A3:**
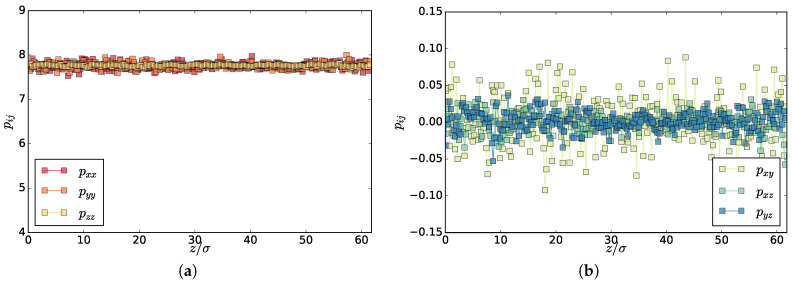
Diagonal (**a**) and off-diagonal (**b**) components of the pressure tensor for $T_{h}=3.0$.

We computed the second (virial) part of the pressure tensor (3) as detailed in [19]. That is, the summation goes over particle pairs *n* and *m* connected by vector ${\vec{r}}_{nm}$, which intersects a mid-plane of a slab at given *z*. The full pressure tensor exhibits a flat profile but unfortunately is too noisy (see Figure A3a,b). Therefore, we can not confirm numerically that the asymmetry shown in Figure 4 is present for the whole tensor, nor can we compute reliably the surface tension as the integral of the difference of its tangential and normal components. However, we observe that the system in the steady state is in mechanical equilibrium.

**Figure 5:**
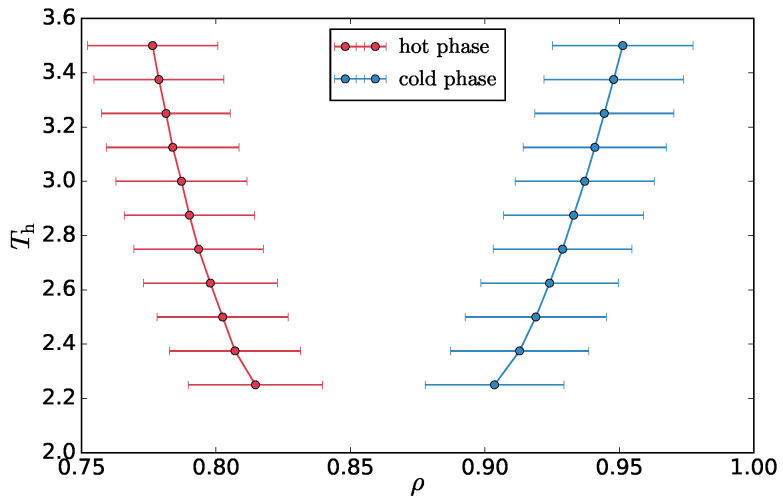
Phase diagram in terms of density. Error bars represent standard deviations of the density in the pure phase.

**Figure A4:**
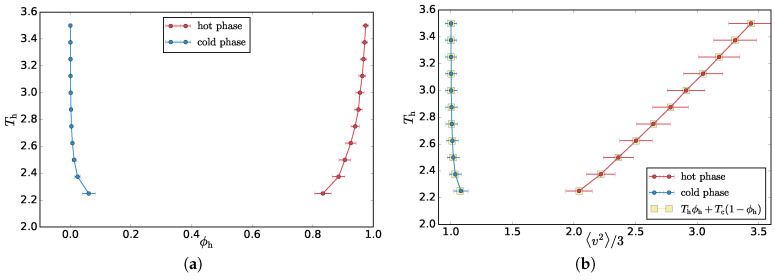
(**a**) phase diagram in terms of number fraction of the hot monomers. Error bars represent standard deviations of the number fraction in the pure phase; (**b**) phase diagram in terms of mean squared velocity (circles). Error bars represent standard deviations of the msv in the pure phase. Yellow squares represent an approximate value of the mean squared velocity in a given phase, given by $T_{h}\phi_{h}+T_{c}\left(1-\phi_{h}\right)$.

We use the plateau values of temperature, density and number fraction, in the average profiles of these quantities, to construct a phase diagram as a function of the $T_{h}$. In Figure 5, we show the phase diagram for the overall density of the two phases and Figure A4a,b shows the number fraction of the hot particles and the msv, respectively.

**Figure 6:**
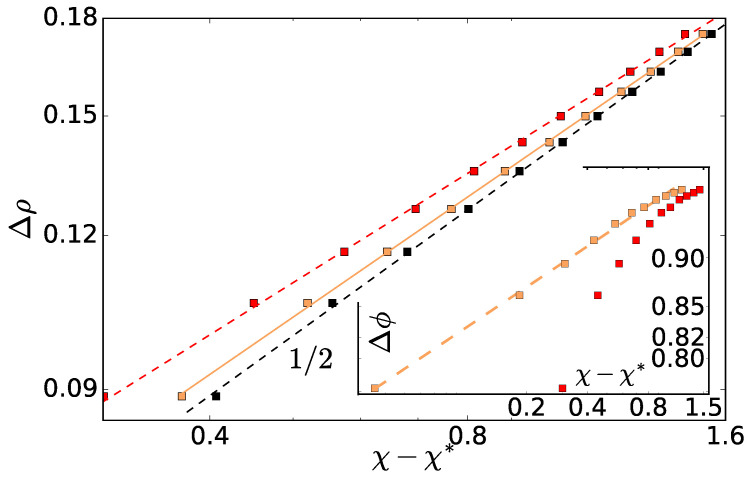
Density difference Δρ as a function of temperature asymmetry χ in log–log scale. Fits of $\Delta \rho =A{\left(\chi -\chi^{*}\right)}^{\beta^{\prime }}$ are shown when (i) (orange) all three parameters *A*, $\chi^{*}$ and $\beta^{\prime }$ are fitted (values $0.143\sigma^{-3}$, 0.36 and 0.47, respectively), (ii) only *A* and $\chi^{*}$ are fitted ($0.140\sigma^{-3}$, 0.32), while $\beta^{\prime }$ is set to 1/2 (black) and (iii) only *A* and $\beta^{\prime }$ are fitted ($0.149\sigma^{-3}$, 0.43) while $\chi^{*}$ is set to 0.43 (red). Note that the *x*-axis $\chi -\chi^{*}$ includes $\chi^{*}$, which is different for the different fits, and, therefore, the data points look like three different sets. Inset: Difference in volume fraction of the hot particles $\Delta \phi_{h}$ as a function of the temperature asymmetry χ. Different curves correspond to different fitting procedures: (i) all three parameters are fitted $A=0.97\sigma^{-3}$, $\chi^{*}=0.69$, $\beta^{\prime }=0.07$ (orange), (ii) $\chi^{*}$ was set to 0.43, but the curve does not exhibit a power law behavior (red). Similarly, the $\Delta \phi_{h}$ data are not consistent with a power law with exponent 0.5 (not shown).

Based on the phase diagrams, one can try to measure an analogue of the equilibrium critical exponent, $\beta_{\mathrm{eq}}$, governing the composition difference as a function of χ. From the density phase diagram (Figure 5), we extract the difference in the phase densities Δρ and examine it as a function of the asymmetry parameter χ. We plot it in Figure 6 in double log scale and fit the data to $\Delta \rho =A{\left(\chi -\chi^{*}\right)}^{\beta^{\prime }}$, to compare it to the behavior of equilibrium phase separation. There, the order parameter (e.g., the density difference) scales with interaction strength with the exponent $\beta_{\mathrm{eq}}=0.3285$ from the Ising universality class, and $\beta_{\mathrm{eq}}=1/2$ is found for a system in the mean-field approximation. The best fit of our data gives A=0.14, $\chi^{*}=0.36$ and $\beta^{\prime }=0.47$. Within our precision, we could not get too close to the critical point and therefore our exponent $\beta^{\prime }$ shows the mean-field behavior. This is typical for polymeric blends due to the extensive number of contacts for each chain (proportional to $N^{1/2}$), which effectively extend the interaction range, and the Ising behavior is observed only when the correlation length becomes much larger than the polymer gyration radius [20]. The critical activity ratio $\chi^{*}=0.36$ is a bit lower than the one found by entropy production $\chi^{*}=0.43$; however, comparably good fits can be achieved when only parameters *A* and $\beta^{\prime }$ are fitted with $\chi^{*}$ set to 0.43 or when $\beta^{\prime }$ is set to 1/2 and *A* and $\chi^{*}$ are fitted (see Figure 6 for details). However, these estimates of the critical exponent and $\chi^{*}$ should be taken with a grain of salt. Firstly, the density difference fluctuations are of the order of $0.02\sigma^{-3}$, which is relatively high. Secondly, the choice of the order parameter should have no effect, apart from a different numerical sensitivity on the value of the exponent $\beta^{\prime }$. However, for example, when we take the difference of the volume fraction of the hot particles as the order parameter, we could not extract any definite range of the exponent (Inset of Figure 6). On the other hand, our finite-size scaling analysis gives some additional support that we see $\beta^{\prime }$ close to the mean-field exponent $\beta_{\mathrm{eq}}$. Certainly, a more detailed work is necessary to determine these and other exponents more precisely. This, however, is quite complicated in the present case as the effective interactions are asymmetric i.e., hot–hot pairs behave differently than cold-cold ones. In equilibrium phase separation, such cases are treated by the “field mixing” [21], which relies on the equilibrium statistical mechanics inapplicable to the present case.

### Finite Size Effects

As a first step, we want to validate the method to determine the critical temperature asymmetry using the crossover of the entropy production detailed in [10]. There, only the entropy production of systems of M=500 and M=1000 chains was compared to get the extent of the finite size effects. The entropy production (per hot particle) $\dot{S}$ in steady state is a sum of the contribution for each reservoir. For reservoir *i*, it is the net heat flux to the reservoir per hot particle $3\zeta (T_{i}^{\mathrm{eff}}-T_{i})$ divided by the temperature of the reservoir $T_{i}$ per unit time. The total entropy production of the system per hot particle then reads

(5) $$\frac{\dot{S}}{3\zeta }=\frac{T_{h}^{\mathrm{eff}}}{T_{h}}+\frac{T_{c}^{\mathrm{eff}}}{T_{c}}-2.$$

**Figure 7:**
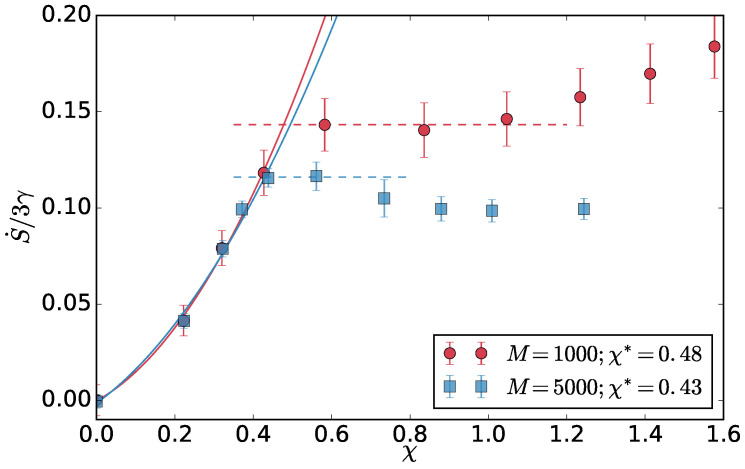
Entropy production per hot particle $\dot{S}$ as a function of χ. The $\chi^{*}$ shown in the legend is extracted as a crossover between the quadratic and constant regime.

We measure the effective temperatures in the simulations and calculate the $\dot{S}$ as above. The entropy production per particle grows with χ, until the two-dimensional interface develops in the system and then it plateaus (drops in the infinite system size limit). The $\chi^{*}$ is obtained as the crossover point between these two regimes. Here, in Figure 7, we compare two system sizes M=1000 and M=5000.

To validate the method, we extract the $\chi^{*}$ using also the standard technique of the fourth order cumulant $U_{n}$ of the density distribution in sub-boxes and compare the results of the two system sizes. The cumulant

(6) $$U_{n}\left(\chi \right)=\frac{\langle n^{-3}\sum_{i}{\left(\rho_{h}^{i}-{\bar{\rho }}_{h}\right)}^{4}\rangle }{{\langle n^{-3}\sum_{i}{\left(\rho_{h}^{i}-{\bar{\rho }}_{h}\right)}^{2}\rangle }^{2}},$$

where $\rho_{h}^{i}$ is the density of the hot particles in *i*-th sub-box out of total $n^{3}$ equal sub-boxes covering the system, ${\bar{\rho }}_{h}$ is the average density of the hot particles (in our case ${\bar{\rho }}_{h}=0.425\sigma^{-3}$) and the brackets 〈…〉 denote average over the different conformations of the system in the steady state. We used also the density of the cold particles with the same results (not shown).

**Figure 8:**
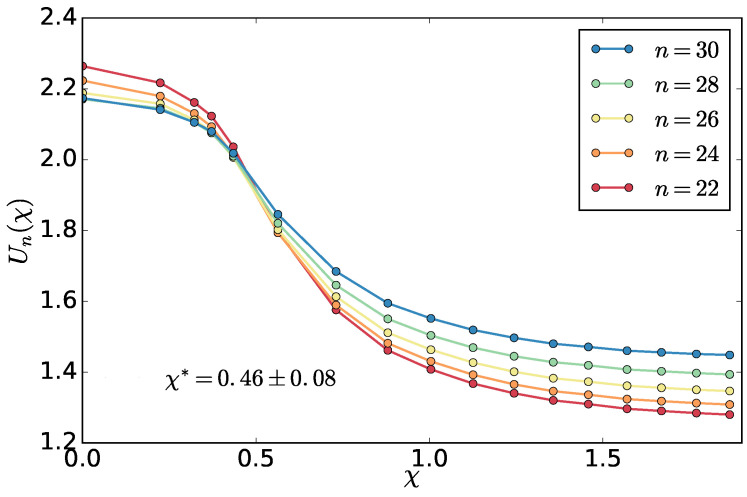
Fourth order cumulant by Equation (6) as a function of the asymmetry χ.

While the entropy production is affected by the choice of the fitting range above the transition point, which is affected by the finite size effects, the cumulant method relies on a proper choice of the value of *n*, which determines the linear size of the sub-box b=L/n. This is to be larger than the correlation length and smaller than the overall system size, so the density fluctuations are not influenced by the fixed ratio of hot and cold particles in the system. As we don’t know these lengths a priori, we try a range of sub-box sizes for the system with M=1000 chains and look at the cumulants crossings. The average crossing point represents an estimate of the critical point and standard deviation an estimate of the error. We select a range of sub-box sizes that gives a result found from the entropy production $\chi^{*}=0.5\pm 0.12$ and use the same sub-box sizes to extract the critical point for the larger M=5000 system. The result ($\chi^{*}=0.46\pm 0.08$ Figure 8) is consistent within the error bars with the result found with the entropy production method. Relatively small sub-box sizes were used as, with our brute-force MD simulations, we could not get too close to the critical point, and, therefore, the correlation length is also limited.

The location of the crossings of the cumulants gives support to the values of the exponent $\beta^{\prime }$ found above. It takes place around their value of about $U_{n}^{*}≃2.0$, which is somewhat closer to the mean-field value of the equilibrium phase transition $U_{n}^{\mathrm{MF}}={\left[\Gamma \left(1/4\right)\right]}^{4}/8\pi^{2}≃2.188$ [22] than the value for the Ising universality class $U_{n}^{\mathrm{Ising}}≃1.59$ [23]. The exponent $\beta^{\prime }$ is also closer to the mean-field value than the one for the Ising as discussed above. This is expected as we could not get very close to the critical point.

**Figure 9:**
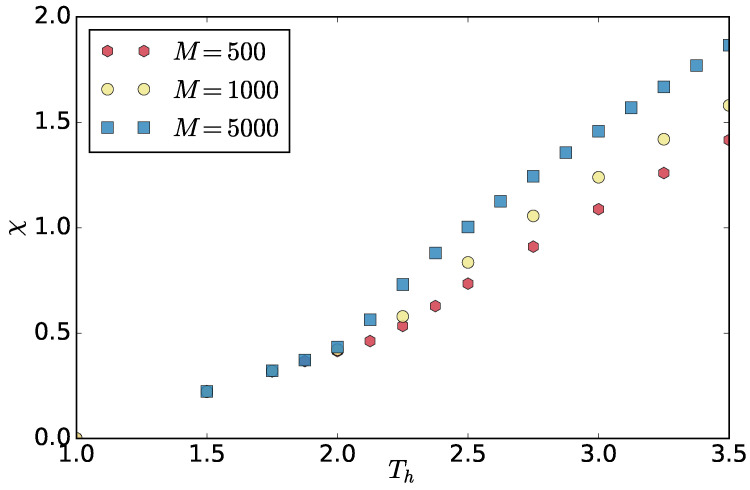
The asymmetry χ as a function of the system size and thermostat temperature $T_{h}$.

The different values of $\chi^{*}$ for different system sizes are the consequence of the fact that χ itself depends on the system size in the separated state. This is due to the fact that the interface represents a finite fraction of the finite system, the effective temperatures of the hot and cold components depend on the fraction and therefore on the system size. This is clearly visible in Figure 9, where one also sees that, in the mixed state, χ does not depend on system size.

Therefore, the cumulant technique represents only an estimate of the critical point for a given finite size system that determines the effective temperatures in the separated case. How the effective temperatures depend on system size remains an open question.

## 3. Capillary Waves and Interfacial Tension

In equilibrium phase separated system, one can extract the interfacial tension from the capillary wave spectrum of the interface [24,25,26]. The capillary wave Hamiltonian is quadratic in the interface height gradient under the assumption of small gradients (no overhangs and bubbles). Thus, in Fourier space, it is diagonal

(7) $$H_{\mathrm{cw}}=\frac{\gamma L^{2}}{4}\sum_{n,m}\left(A_{nm}^{2}+B_{nm}^{2}\right)q_{nm}^{2},$$

where γ is the interfacial tension, *L* is the system size and *A*s and *B*s are the amplitudes corresponding to the mode $q_{nm}$. For more details, see [25,26] and the Appendix A. Using the equipartition theorem, every modes carries on average energy of $\beta^{-1}/2$ and therefore the capillary wave spectrum is quadratic in inverse *q*,

(8) $$L^{2}\langle C^{2}\rangle =\frac{2}{\gamma \beta q^{2}},$$

where we use the notation from [25]: $C^{2}=\left(A^{2}+B^{2}\right)/2$ and we drop the subscripts n,m.

In our out-of-equilibrium system, we can not write a Hamiltonian or use the equipartition theorem for capillary waves, but we can measure the spectrum of the interfacial fluctuations. The method to do so can be directly generalized for the present nonequilibrium case and is summarized in the Appendix A. As detailed there, for analysis purposes, we examine the interface subdivided into $n_{B}\times n_{B}$ equal sized ($L/n_{B}$) blocks in *x*- and *y*-directions. This division into blocks filters out all wave vectors larger than $2\pi n_{B}/L$.

**Figure 10:**
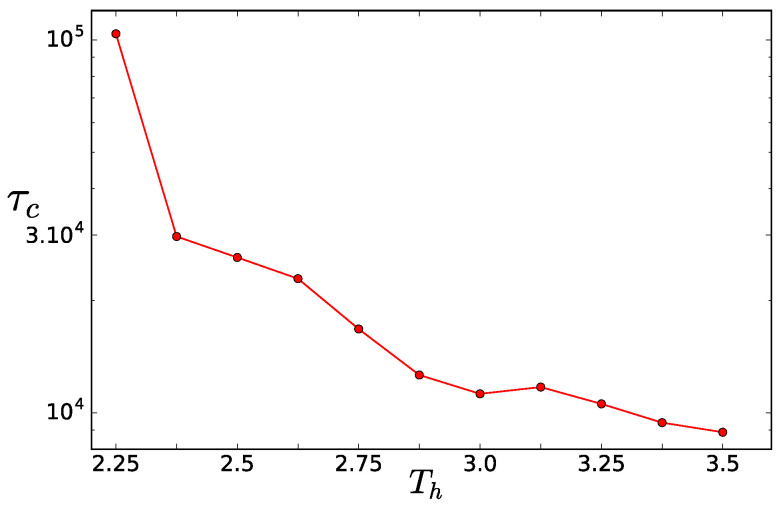
Correlation time of the slowest capillary mode as a function of the hot thermostat temperature.

To obtain a correct spectrum, we have to take conformations separated by a time span larger than the correlation time of the slowest capillary mode (n=0 and m=1, corresponding to $\vec{q}=\left(0,2\pi /L\right)$). We analyzed the interface position for the mode by computing its autocorrelation function

(9) $$c\left(t\right)=\frac{\langle C_{01}\left(t\right)C_{01}\left(0\right)\rangle -\langle C_{01}^{2}\rangle }{\langle C_{01}^{2}\rangle -{\langle C_{01}\rangle }^{2}}$$

and fitting it with the exponential $\exp(-t/\tau_{c})$. The correlation time $\tau_{c}$ as a function of the thermostat temperature is plotted in Figure 10. To get the spectrum, we only used a subset of all the configurations, i.e., configurations separated by $3\times 10^{4}\tau$, which is larger than all $\tau_{c}$ except for $T_{h}=2.25$. Contrary to [25], the correlation time decreases with the increasing asymmetry characterized by $T_{h}$.

**Figure 11:**
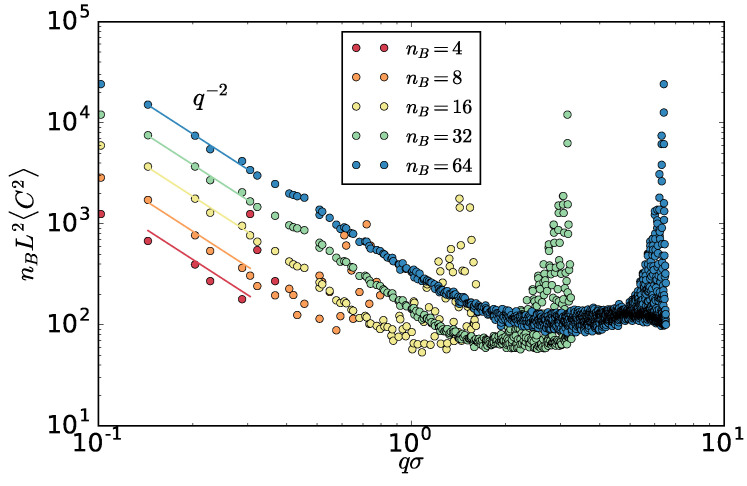
Spectrum of the capillary waves for $T_{h}=3.0$ for different block divisions $n_{B}$. For clarity, the amplitude $\langle C^{2}\rangle$ is multiplied by $n_{B}$. Lines correspond to fits of the prefactor in (8). The interfacial stiffness is obtained as an average of values from $n_{B}=16,32$ and 64. These values differed by 0.015.

**Figure 12:**
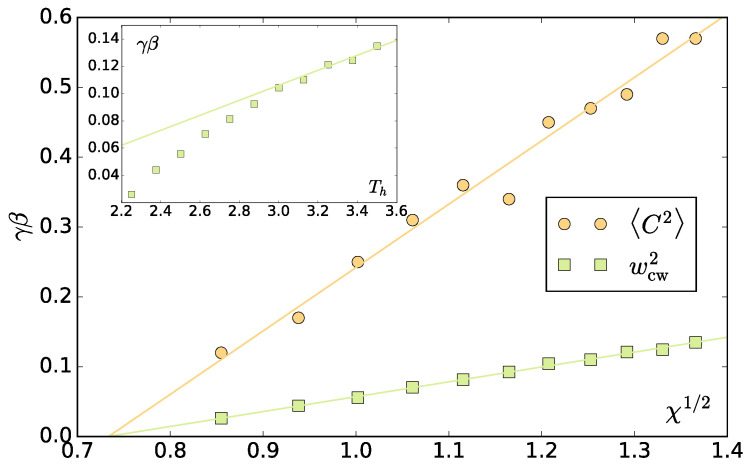
Interfacial stiffness as a function of $\chi^{1/2}$ extracted from capillary amplitude scaling (circles) and mean square width analysis (squares). Lines in main plot are linear fits, intersecting at γβ=0, which gives $\chi^{*}=0.54$. Inset: plotted as a function of $T_{h}$. The line in the inset is a linear guide to the eye.

Surprisingly, we find that the amplitude is, as in equilibrium, proportional to $q^{-2}$ and independent from the block number $n_{B}$ (Figure 11). This allows us to use the interfacial fluctuations as a mechanistic definition of the interfacial stiffness γβ. One way to do this is extracting the γβ from a fit of (8) to the spectrum. To ensure we are not affected by the correlations in the slowest mode, we additionally left out the slowest mode from the fit. In fact, this does not change the resulting values except for the correlated case of $T_{h}=2.25$. In Figure 12, the resulting γβ as a function of $T_{h}$ and χ are shown.

We stress that, in equilibrium, the two symbols of interfacial stiffness γβ represent the product of the interfacial tension and the inverse temperature. The main result of [14,15] is that, for the vectorial activity separation, γ is the surface tension consistent with the definition (4), which is negative, and β corresponds to the active work dissipated to the environment, which is also negative. While the negative surface tension amplifies fluctuations, eventually leading to a surface rupture, the actively swimming particles oriented towards the surface push towards the dense phase and reform the surface again on the expense of their active work. This gives rise to the positive stiffness that governs the surface fluctuations. In the present scalar case, we cannot conclude the signs of γ and β separately, but we do observe a positive interfacial stiffness and also an equilibrium-like spectrum. Although β can be related to the excess energy present in the system due to activity, there is no orientational order at the interface in the scalar case. We hypothesize that the velocity anisotropy in Figure 4 could play a role.

An alternative way to obtain the interfacial stiffness is from the mean squared interfacial width. Because of the $q^{-2}$ dependence, the present non-equilibrium interface suffers from broadening similar to its equilibrium counterpart: The mean square width of the interface position due to capillary waves is given by

(10) $$w_{cw}^{2}=\langle h^{2}\rangle -{\langle h\rangle }^{2}=\sum_{n,m}\langle 2C^{2}\rangle .$$

Using (8), this can be written as an integral over wave vectors in polar coordinates

(11) $$w_{cw}^{2}=\frac{1}{2\pi \beta }\int_{\Lambda_{\min}}^{\Lambda_{\max}}\frac{dq}{\gamma q}=\frac{1}{2\pi \gamma \beta }\ln\left(\frac{\Lambda_{\max}}{\Lambda_{\min}}\right),$$

where the cutoff $\Lambda_{\min}=2\pi /L$ is set by the smallest possible wave vector in a finite system of size *L*. The cutoff $\Lambda_{\max}=2\pi /a$ defines a length *a* below which the approximation of smooth interface with small gradients is invalid. This length scale is unknown, but one can investigate the width at different resolutions by setting $a=cL/n_{B}$. This gives

(12) $$w_{cw}^{2}=\frac{1}{2\pi \gamma \beta }\ln n_{B}/c,$$

where *c* is a constant.

Similarly to [25], one could extract the interfacial stiffness also from the scaling of $w_{cw}$ with the system size. We do not use this method here because the χ depends on the system size (shown in Figure 9) and one would need to include this yet unknown dependency. For completeness, we provide in the Appendix A an adaptation of the method for the present case of asymmetric phases and discuss where the χ(L) dependence comes into play.

**Figure 13:**
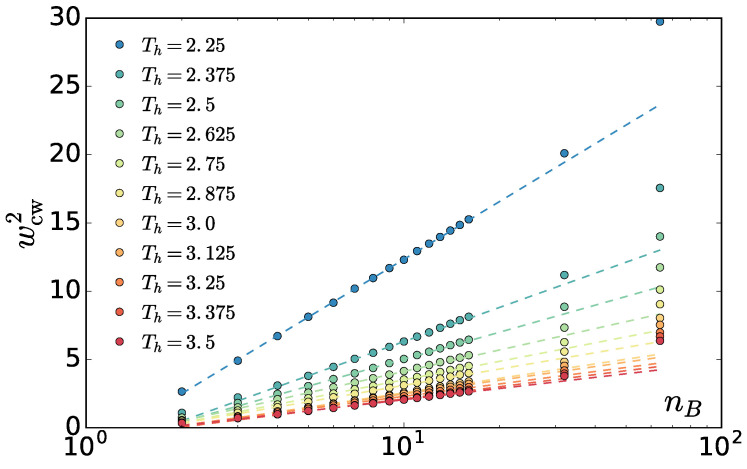
Mean square apparent interface width as a function of $n_{B}$ in log scale for various $T_{h}$. Dashed lines are fits of Equation (A11) to range $n_{B}\in \left[5,9\right]$, although the log-linear regime extends well up to $n_{B}=16$.

In practice, using (12), we measure the interface width as a function of the $\ln n_{B}$ and extract the slope in the log-linear regime (Figure 13).

We do this for each temperature asymmetry and extract the dependence of the stiffness γβ on χ (Figure 12).

The different methods yield different values of interfacial stiffness. This is also observed in equilibrium systems, where the source of this discrepancy is not completely clear. It is attributed to correlation effects between subsequent interface snapshots, or the different methods inherently producing different results [25]. The former reason seems unlikely for our case, as in our system the correlation time decreases with χ (Figure 10). The latter case is more likely. The phases in our system exhibit different densities at different χ, which is an additional complication avoided in the standard work [25]. Although we are not aware of how this difference should be incorporated at the level of $w_{cw}$, it can not be denied that it has an effect on the resulting value of γβ, which would agree with the fact that the discrepancy is growing with χ. Nevertheless, the trends for both methods are the same: the interfacial stiffness scales linearly with $\chi^{1/2}$, which is again reminiscent of the equilibrium case. This is more visible for the $w_{\mathrm{cw}}^{2}$ method, which is less noisy as the values are obtained from a two parameter fit (*c* and γβ) instead of one in the amplitude case.

From the linear fit of γβ versus $\chi^{1/2}$, we can extract another estimate of the critical asymmetry as $\gamma \beta (\chi^{*})=0$. Both methods give the same value $\chi^{*}=0.54$, which gives credence to our analysis. This value of $\chi^{*}$, agrees, within errorbars, with the one obtained from the cumulant method above ($\chi^{*}=0.46\pm 0.08$). Additionally, in Figure A6, we compare the present interfacial stiffness dependence with one obtained for a smaller system with M=1000. The values and the location of $\chi^{*}$ obtained from the width analysis agree very well with the ones found for the larger system.

**Figure 14:**
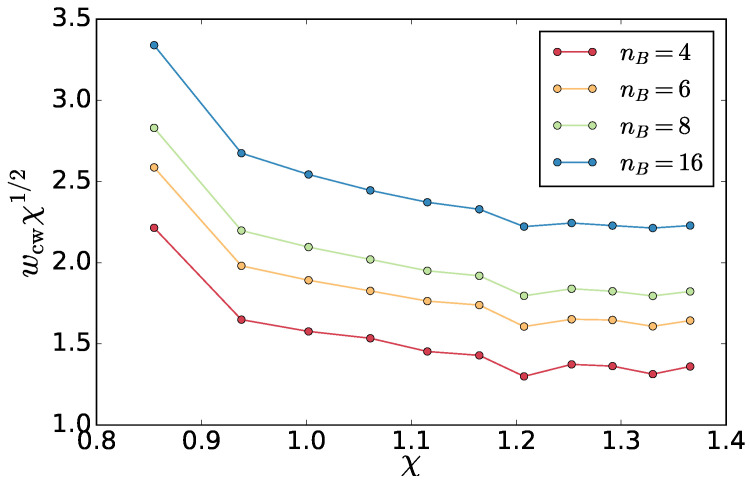
Interfacial width from Figure 13 multiplied by $\chi^{1/2}$ as a function of the asymmetry χ for various block numbers $n_{B}$.

**Figure A5:**
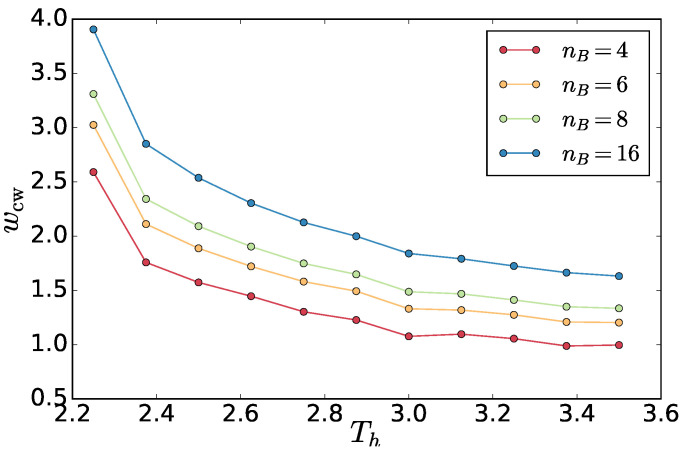
Interfacial width from Figure 13 as a function of the temperature of the hot thermostat $T_{h}$ for various block numbers $n_{B}$.

**Figure A6:**
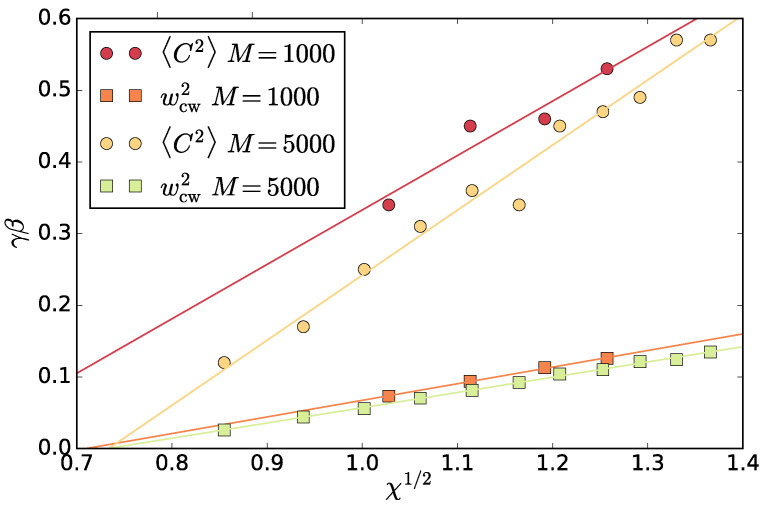
Interfacial stifness γβ as a function of $\chi^{1/2}$ extracted from capillary amplitude scaling (circles) and mean square width analysis (squares) for two system sizes. Lines are linear fits. The values of $\chi^{*}$ for M=1000 system are $\chi^{*}=0.5$ for width analysis, which agrees very well with the value for M=5000, and $\chi^{*}=0.32$ for the capillary amplitude analysis. The discrepancy between the two methods can be attributed to the fact that we have only four data points for M=1000, which makes the fit of the more noisy capillary amplitude data unreliable. Moreover, additional finite size effects can play a role.

From the analysis above and Figure 13, we also obtained the width of the interface as a function of the temperature asymmetry χ. This is plotted in Figure 14 for various block numbers $n_{B}$ from the linear regime of Figure 13 (see also Figure A5 in the Appendix B for plot with respect to $T_{h}$). The width values depend on $n_{B}$, but the profile is universal, and, for large χ, it approaches $w∼\chi^{-1/2}$ dependence. This is the same as the dependence found for equilibrium phase separation with two polymer species characterized by Flory parameter χ. The latter can be understood by a simple scaling argument: the energy of a loop of polymer A in the B phase gets an energy penalty of $N_{\mathrm{loop}}\chi k_{B}T$. In equilibrium, this must be of the order of $k_{B}T$ and therefore we have $N_{\mathrm{loop}}∼1/\chi$. As the width of the interface is proportional to the size of this loop $w∼N_{\mathrm{loop}}^{1/2}$, we get the result above [27]. In the non-equilibrium case, we hypothesize that a typical magnitude of the fluctuations of the entropy production gives rise to a loop size as $N_{\mathrm{loop}}∼1/\chi$, which gives the same behavior of the interface width as in equilibrium.

## 4. Discussion

In this work, we examine a scalar active-passive (two-temperature) polymer mixture that for high-enough temperature asymmetry exhibits non-equilibrium phase separation. At first, we show that the system has low sensitivity to the chosen friction ζ when studied using the effective temperatures in form of the asymmetry parameter χ of the two components. Still, a small variation of the order parameter with ζ remains, which poses a question of the existence of a truly unifying description of systems with different frictions.

Using aligned, well-separated systems, we extract the spatial profiles of kinetic and static properties such as mean square velocities and densities. In contrast to the equilibrium behavior, analysis of the pressure tensor profile reveals that its kinetic part can contribute to the interfacial tension. Based on the profiles, we construct phase diagrams that can further be used to extract the critical exponents governing the transition. While our preliminary study suggests the related critical exponent $\beta^{\prime }$ to be between the equilibrium mean-field value 1/2 and the Ising universality class 0.3285, its exact value and the value of other critical exponents require a more detailed study. However, the complication lies in the asymmetry of the effective interactions. The equilibrium approach is to study the transition in a grand-canonical ensemble along with the use of histogram re-weighting and field mixing methods. Here, equilibrium statistics is violated, and new analogous methods would first have to be developed to allow for an accurate universality classification of the transition.

We determine the critical asymmetry $\chi^{*}$ in our system using various methods: (i) density based fourth order cumulant; (ii) extrapolation of critical density difference; (iii) analysis of capillary waves amplitudes and (iv) interfacial width. We find consistent results with the entropy production crossover method introduced in [10].

We perform the analysis of the interfacial fluctuations and find again scaling of the interfacial stiffness and interfacial width χ similar to equilibrium mixtures with lower critical solution temperature. The works [14,16] with vectorial activity assumed the equipartition theorem and therefore also the equilibrium-like capillary waves spectrum. Here, we explicitly construct the spectrum for scalar activity and find its equilibrium like nature. The very fact that we obtain equilibrium-like spectrum suggests an existence of some sort of equipartition theorem source, which remains unknown. It is interesting that both the vectorial and scalar activity models exhibit similar properties of the interfacial fluctuations. This suggests that a unifying description of these fundamentally different out-of-equilibrium systems might be possible. Therefore , determining the origin of the equilibrium-like fluctuations certainly deserves attention in future. A step in this direction could be to find out whether the interfacial stiffness derived from the capillary waves and related interfacial tension are relevant in the nucleation kinetics.

## Figures and Tables

**Table:** 

	
(**a**)	(**b**)

**Table:** 

	
(a)	(b)

**Table 1:** Mean squared gyration radius of hot and cold chains in different regions for $T_{h}=3.0$. Relatively large standard deviation for the hot chains in the cold region is caused by their small number fraction.

Region	$R_{g}\left(\sigma^{2}\right)$ of Hot Chains	$R_{g}\left(\sigma^{2}\right)$ of Cold Chains
hot	10.6±0.1	10.8±0.5
cold	8.1±3.3	10.1±0.2
transition	10.0±0.1	10.5±0.1

**Figure A1:**
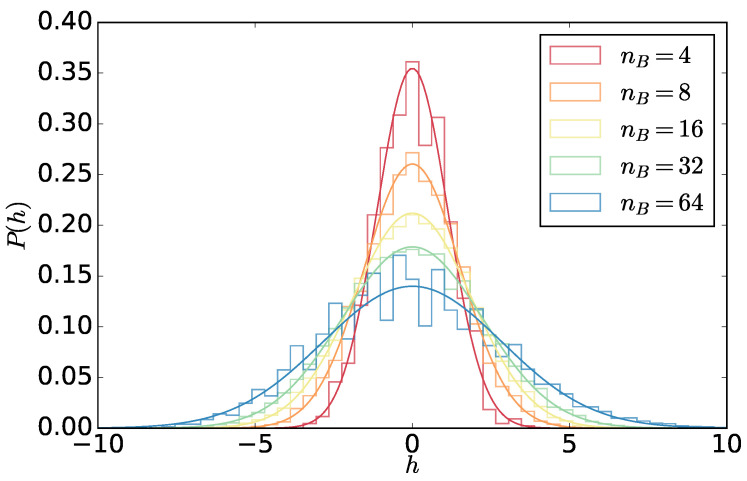
Centered distributions of interface position for $T_{h}=3.0$ for various $n_{B}$.

**Table:** 

	
(**a**)	(**b**)

**Table:** 

	
(**a**)	(**b**)

## Appendix A. Capillary Waves Theory and Analysis

The equilibrium capillary waves Hamiltonian in the small interface gradient limit ∇h≪1 is

(A1) $$H_{\mathrm{cw}}=\gamma \int_{0}^{L}\int_{0}^{L}{\left(\nabla h\right)}^{2}dxdy.$$

Using the Fourier transform,

(14) $$\begin{array}{c} A_{nm}=\frac{1}{L^{2}}\int_{0}^{L}\int_{0}^{L}h\left(x,y\right)\cos\left(\vec{r}\cdot {\vec{q}}_{nm}\right)dxdy,\\ B_{nm}=\frac{1}{L^{2}}\int_{0}^{L}\int_{0}^{L}h\left(x,y\right)\sin\left(\vec{r}\cdot {\vec{q}}_{nm}\right)dxdy, \end{array}$$

the interface position *h* at $\vec{r}=\left(x,y\right)$ is given by

(A3) $$h\left(x,y\right)=A_{00}+\sum_{n,m}A_{nm}\cos\left(\vec{r}\cdot {\vec{q}}_{nm}\right)+B_{nm}\sin\left(\vec{r}\cdot {\vec{q}}_{nm}\right).$$

Using the Fourier series, the Hamiltonian gets the form of Equation (7).

To analyze the capillary waves, we follow the block analysis method [25,28] as detailed in [26] and adapt it to our situation. This method was demonstrated to be accurate and robust in studies of equilibrium interfaces and interfacial tension. We briefly summarize it below.

In what follows, we always use the density of the cold monomers, but equivalently one could use hot monomers instead. Firstly, for each interface i=1,2, we determine its global position $z_{i}$ and width $w_{i}$. This is done by dividing the box into 100 slabs parallel to the interface, computing average density (of cold monomers) of a given slab and fitting the density profile (A4) for each interface. The fitted density profile is assumed to be the one of the equilibrium phase transition

(A4) $$\rho^{\mathrm{id}}\left(z\right)=\frac{\rho_{c}+\rho_{h}}{2}+\frac{\rho_{c}-\rho_{h}}{2}\tanh\left(\frac{z-z_{i}}{w_{i}}\right),$$

which agrees with the data well. Since a priori we do not know the densities of the cold monomers in the hot and cold phases, i.e., $\rho_{h}$ and $\rho_{c}$, we take these as fit parameters too and we find that their values are well reproducible for all studied conformations.

Then, we divide the simulation box into blocks of size $L/n_{B}\times L/n_{B}\times l_{i}$, centered around the interface position $z_{i}$, with the height of the block $l_{i}=2\alpha w_{i}$ i.e., the *z* coordinates of block ends are $z_{i}^{\pm }=z_{i}\pm \alpha w_{i}$. The value of the parameter α>1 is chosen as a compromise between the aim to include all the interface undulations and that the blocks of the two interfaces are not overlapping significantly. Typically, for our system sizes, this is satisfied by a value of α=2, but, as we checked, the result trends are insensitive to this choice, which is true also for the equilibrium simulations in Ref. [26].

The height of the interface h(x,y) in the block located at (x,y) is then found from the density profile ρ(z) within the block by Gibbs-like condition:

(A5) $$\int_{z_{1}^{-}}^{h}\rho \left(z\right)dz-\rho_{h}\left(h-z_{1}^{-}\right)=\rho_{c}\left(z_{1}^{+}-h\right)-\int_{h}^{z_{1}^{+}}\rho \left(z\right)dz,$$

i.e., the areas spanned by the density profile and the densities of the hot and cold phases are set to be equal. Here, we assumed, without loss of generality, that the hot (less dense) phase is located below the interface i=1$\left(z<z_{1}\right)$ and the cold phase is present above $\left(z>z_{1}\right)$. From (A5), the interface height is

(A6) $$h=\left(n_{c}/{\left(L/n_{B}\right)}^{2}+\rho_{h}z_{1}^{-}-\rho_{c}z_{1}^{+}\right)/\left(\rho_{h}-\rho_{c}\right),$$

where $n_{c}$ is the number of cold monomers in the slab. Similar conditions and expressions for the height is obtained for the other interface and one gets complete h(x,y) for both interfaces. As presented in [26], this construction is independent of the specific profile form within the block and works well even for noisy profiles that are arising from small number of particles within blocks. We Fourier transform each interface *h* using Equation (A2) and calculate the amplitudes as a function of the wave vector *q*.

### Interface Broadening for Unequal Densities

The width observed in a simulation is a combination of the the intrinsic width and the width due to capillary waves. In our case, however, the standard convolution approximation derived in [24] has to be amended due to different densities of the hot and cold phase.

The convolution approximation combines the intrinsic width of the ideal profile (A4) with a Gaussian fluctuations due to capillary waves to get the apparent profile

(A7) $$\rho^{\mathrm{app}}\left(z\right)=\int_{-\inf}^{\inf}\rho^{\mathrm{id}}\left(z-h\right)P\left(h\right)dh,$$

where $P\left(h\right)=\exp\left(-h^{2}/2w_{cw}^{2}\right)/{\left(2\pi w_{cw}^{2}\right)}^{1/2}$. As we show in Figure A1 the distribution of the local interface position is indeed a Gaussian.

Following [24], to perform the integral it is useful to approximate the tanh with the error function of the same in the midpoint, which for our case is

(A8) $$\rho^{\mathrm{id}}\left(z\right)\approx \frac{\rho_{c}+\rho_{h}}{2}\left(1+\frac{\rho_{c}-\rho_{h}}{\rho_{c}+\rho_{h}}\mathrm{erf}\left(\frac{\sqrt{\pi }\left(z-z_{i}\right)}{2w_{0}}\right)\right).$$

The apparent interfacial width is defined from the maximum slope of the apparent density profile by

(A9) $$\frac{1}{2w}=\frac{2}{\rho_{c}+\rho_{h}}{\left.\frac{d}{dz}\rho^{\mathrm{app}}\left(z\right)\right|}_{z=z_{i}}.$$

The result

(A10) $$w^{2}=A{\left(\chi \right)}^{2}\left(w_{0}^{2}+\frac{\pi }{2}w_{cw}^{2}\right)$$

differs from the original convolution approximation only by a factor $A{\left(\chi \right)}^{2}={\left[\left(\rho_{c}+\rho_{h}\right)/\left(\rho_{c}-\rho_{h}\right)\right]}^{2}$. Using the Equation (12), we get the result

(A11) $$w^{2}=A{\left(\chi \right)}^{2}w_{0}^{2}+\frac{A{\left(\chi \right)}^{2}}{4\gamma \beta }\ln\left(L/a\right),$$

where the correction factor $A\left(\chi \right)=\left(\rho_{c}+\rho_{h}\right)/\left(\rho_{c}-\rho_{h}\right)$ is the inverse relative difference in the density of the cold monomers in the cold and hot phase, which depends on the temperature asymmetry χ. The value of the correction factor for each temperature can be read out from the phase diagrams of density and number fraction ($\rho_{i}=\left(1-\phi_{h}\right)\rho$, with ρ being the overal density of the phase i=c,h). As mentioned above and in Figure 9, χ itself depends on the system size. This dependence causes the factor A(χ) to be *L* dependent and hence it is crucial to know it explicitly. Then, varying the system size *L*, one could extract the interfacial stiffness γβ. Note that this can not be used to obtain the intrinsic width $w_{0}$ as this is convoluted with *a* by the constant *c* in (12) as pointed out in [26].

## Appendix B. Additional Profiles, Phase Diagrams and Analysis Details

### Appendix B.1. Additional Profiles

Profile of hot particles number fraction $\phi_{h}$ is in Figure A2. Components of pressure tensor (3) are in Figure A3.

### Appendix B.2. Additional Phase Diagrams

Phase diagrams in terms of number fraction and msv are shown in Figure A4a,b, respectively.

### Appendix B.5. Thermostat Anisotropy

As is standard in simulation packages, our Langevin thermostats draw their random force direction form a cube, i.e., each of the three components is drawn uniformly from [−1,1] interval. This is indeed non-physical as at the microscopic level the system should not know about the laboratory coordinates. Moreover, this microscopic anisotropy could in principle manifest itself on the macroscopic scale. Because the phase separation arises as the consequence of the different thermostat temperatures, one should find out if the anisotropy changes the critical temperature or the shape of the interface.

Therefore, we ran additional simulations with an isotropic thermostats that generated forces from a unit sphere. As we checked by monitoring the number density based order parameter [10], this did not have an influence on the critical temperature ratio within our precision. In addition, above the critical temperature ratio, the interface aligned with the box irrespective of what thermostat was used. If a different hot-cold fraction was used, a droplet state could be observed, which could be slightly anisotropic. Let us emphasize that this is not the case for equilibrium phase separation as there the system behavior is governed by the difference in the (isotropic) interactions and the anisotropic thermostat could only cause anisotropic shape *fluctuations*, but, on average, the droplet is isotropic.
